# The Risk of Breast Cancer in Women Using Menopausal Hormone Replacement Therapy in Taiwan

**DOI:** 10.3390/ijerph13050482

**Published:** 2016-05-11

**Authors:** Jui-Yao Liu, Tzeng-Ji Chen, Shinn-Jang Hwang

**Affiliations:** 1 Department of Family Medicine, Taipei Veterans General Hospital, No.201, Sec. 2, Shi-Pai Road, Taipei 11217, Taiwan; tjchen@vghtpe.gov.tw (T.-J.C.); sjhwang@vghtpe.gov.tw (S.-J.H.); 2 School of Medicine, National Yang-Ming University, No.155, Sec. 2, Linong Street, Taipei 11217, Taiwan

**Keywords:** menopausal hormone replacement therapy, breast cancer, birth-cohort effect, National Health Insurance Research Database, Asian population

## Abstract

Menopausal hormone replacement therapy (HRT) increases the risk of breast cancer in Western countries; however, there are fewer reports from the Asian population, which has a lower incidence of breast cancer. A population-based retrospective cohort study was conducted by analyzing longitudinal National Health Insurance claim data of a 200,000-person national representative cohort. A total of 22,929 women aged ≥45 years in 1997 without previous diagnosis of breast cancer were enrolled and stratified into two birth cohorts born before or after 1933. HRT prescriptions were traced in outpatient data files and incident breast cancer cases were identified from 1997 to 2004. The Cox proportional hazards model was used to analyze breast cancer hazard ratio (HR). HRT users were censored after they discontinued HRT. The results showed that women born during 1933–1952 had a twofold increased risk of breast cancer (HR = 2.10, 95% CI = 1.47–3.00) compared with women born before 1933, when adjusted for HRT use. When adjusted for the birth-cohort difference, HRT users had significantly increased breast cancer HR *versus* non-users after four years of use (adjusted HR = 1.48, 95% CI = 1.03–2.13); the HR further increased to 1.95 (95% CI = 1.34–2.84) after eight years of use. In conclusion, a longer duration of current HRT use was associated with a higher risk of breast cancer independent of the birth-cohort difference.

## 1. Introduction

Both randomized controlled trials and observational studies conducted in Western countries have shown that menopausal hormone replacement therapy (HRT) increases the risk of breast cancer [1,2,3,4,5,6], but there are fewer reports from the Asian population [7,8,9]. HRT may exert heterogeneous effect to different races [10]. Asian women have a lower incidence of breast cancer than do Caucasian women [11,12,13], probably due to their relative breast-cancer-protective lifestyles and leaner body size [14,15]. With the on-going Westernization of lifestyles, the incidence of breast cancer is increasing in Taiwan. There is an obvious birth-cohort difference observed, with the elderly generation retaining a low risk of breast cancer, while the younger birth cohort shows higher breast cancer incidence peaking at peri-menopausal ages [16,17]. It remains unanswered whether HRT use or a birth-cohort effect contributes to this phenomenon [18].

Single-payer National Health Insurance (NHI) implemented in Taiwan since 1995 has covered the health care of over 99.9% of the population. In this study, we analyzed a national representative cohort with eight years of longitudinal NHI claim data to investigate whether HRT increased the risk of breast cancer in the Asian population.

## 2. Methods

The National Health Insurance Research Database (NHIRD) comprises NHI reimbursement claim data and registry records collected by the National Health Insurance Administration and processed by the National Health Research Institute for clinical and epidemiological research [19]. All the data files in the NHIRD are de-identified by scrambling the identification codes of both patients and medical facilities to make it impossible to identify any individuals at any level. All the applications for data release were reviewed for approval by the National Health Research Institute [19].

The NHIRD has a 200,000-person national representative cohort sampled from 23,753,407 beneficiaries enrolled in NHI before 2001 by simple random sampling. All the longitudinal inpatient and outpatient claim data from 1997 to 2004 and cancer registry records in the NHI Catastrophic Disease Registry from 1995 to 2004 were retrieved. Women fulfilling the inclusion criteria of being female, alive, aged ≥45 years in 1997, and without a previous diagnosis of breast cancer, were enrolled. A total of 22,929 women fulfilled the inclusion criteria and were further stratified into two birth cohorts—those born from 1933 to 1952 (aged 45–64 years in 1997) and those born before 1933 (aged ≥65 years in 1997). Outpatient claim data from 1 January 1997 to 31 December 2004 were reviewed to identify the date, duration, and regimen of HRT prescriptions, including all estrogen and progestin types in oral or topical forms. The duration of HRT use was calculated only during 1997 to 2004, as there were no outpatient claim files before 1997 in the NHIRD. Women who were prescribed HRT continually for longer than 3 months during 1997 to 2004 were classified as HRT users; the others were classified as non-users. HRT users were followed from the date of commencing HRT until three months after discontinuation of HRT to cover time lag from diagnosis to registration to the National Health Insurance Administration. HRT users were censored after discontinuation of HRT because the risk of breast cancer dissipated after discontinuation of HRT use [3,6,20]. Non-users were followed from 1 January 1997 to 31 December 2004. The incident of breast cancer cases from 1997 to 2004 were identified in the NHI Catastrophic Disease Registry with the diagnostic code of 174 by the International Classification of Disease, Ninth Revision, Clinical Modification (ICD-9-CM). The breast cancer hazard ratio (HR) of HRT users *versus* non-users was analyzed using the Cox proportional hazards model, adjusted by birth cohort (birth after or before 1933). Kaplan–Meier estimates of cumulative breast cancer hazards of the middle-aged cohort were plotted after being stratified by HRT use. Breast cancer incidence was estimated by dividing the number of breast cancer cases by the total observed person-years. Data were analyzed using the PASW statistics 18 software (SPSS Inc. Chicago, IL, USA). *p* values <0.05 (2-tailed) were considered statistically significant.

## 3. Results

### 3.1. Women Aged 45–64 Years (the Cohort Born during 1933–1952)

**Figure 1:**
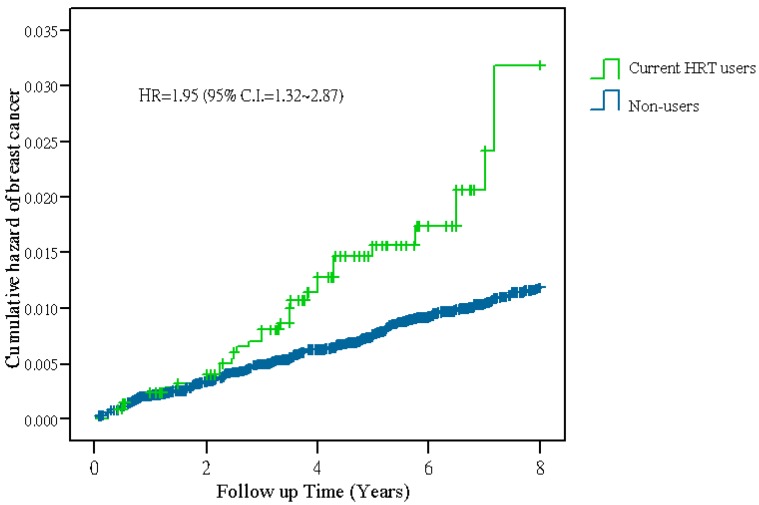
Kaplan-Meier estimates of cumulative breast cancer hazards of the middle-aged cohort, stratified by HRT use.

Among 15,863 women aged 45–64 years on enrollment, 3397 (21.4%) women received HRT with a mean duration of 3.4 years during 1997 to 2004. Of the HRT users, 29.8% received HRT for longer than 5 years. There were 34 incident breast cancer cases in a total of 11,371 person-years of follow-up for the current HRT users (average 3.4 years per person); the breast cancer incidence was 299 cases per 100,000 person-years. Among 12,466 non-users, there were 147 incident breast cancer cases in a total of 96,246 person-years of follow-up (average 7.7 years per person); the incident rate was 153 cases per 100,000 person-years. The breast cancer hazard ratio of current HRT users *versus* non-users was 1.95 (95% CI = 1.32–2.87) by the Cox proportional hazards model (Table 1, Figure 1). Women on estrogen combined with progestin had a significantly higher breast cancer HR than non-users (HR = 2.17, 95% CI = 1.41–3.32), as did those on a sequential combined regimen (HR = 2.4, 95% CI = 1.25–4.61) or on a continuous combined regimen (HR = 1.98, 95% CI = 1.18–3.32). Women taking only estrogen did not have a significantly higher breast cancer HR than non-users (HR = 1.37, 95% CI = 0.64–2.95) (Table 1).

### 3.2. Women Aged ≥65 Years (the Cohort Born before 1933)

Among 7066 women aged ≥65 years, 550 (7.8%) received HRT with a mean duration of 2.6 years from 1997 to 2004. Approximately 16% of the elderly HRT users received HRT for longer than 5 years. There were two incident breast cancer cases in a total of 1412 person-years of follow-up of current HRT users (average 2.6 years per person); the incident rate was 142 cases per 100,000 person-years. Among 6516 elderly non-users, there were 34 incident breast cancer cases in a total of 44,385 person-years of follow-up (average 6.8 years per person); the incident rate was 77 cases per 100,000 person-years. The breast cancer hazard ratio of current HRT users *versus* non-users was 1.85 (95% CI = 0.43–7.90) in the elderly cohort.

### 3.3. Birth-Cohort Effect

By adjusting HRT use, the middle-aged cohort had an approximately twofold increase in the risk of breast cancer compared with the elderly cohort using the Cox proportional hazards model (adjusted HR = 2.10, 95% CI = 1.47–3.00). The birth-cohort risk difference was independent of HRT use.

### 3.4. Duration of HRT Use

In the Cox proportional hazards model adjusted for birth-cohort difference, HRT users had significantly higher breast cancer HR *versus* non-users after four years of current HRT use (adjusted HR = 1.48, 95% CI = 1.03–2.13). The adjusted HR increased to 1.95 (95% CI = 1.34–2.84) after eight years of continual HRT use (Table 2).

### 3.5. Population-Based Risk of Breast Cancer Attributable to HRT in Taiwan

The population-based risk of breast cancer attributable to HRT (PAR) is defined as the proportional reduction in the risk of breast cancer in the population if all HRT exposure was removed. It depends on both the prevalence of the HRT use and the strength of its association (relative risk). The formula is as follows: 

PAR = P_e_ (RR_e_-1)/[1 + Pe (RRe − 1)], where P_e_ is the proportion of HRT use in population, and RR_e_ is the relative risk of breast cancer associated with HRT exposure.

In middle-aged women, there were 1.5 additional cases of breast cancer for every 1000 person-years of long-term HRT use (299 cases per 100,000 person-years in HRT users *versus* 153 cases per 100,000 person-years in non-users). The population-based risk of breast cancer attributable to HRT in the middle-aged cohort was 10.3% supposing that 78.6% of them did not use HRT, with the relative risk of breast cancer as 1, and there was a relative risk of 1.3 for the 15% that used HRT less than 5 years, and a relative risk of 1.9 for the 6.4% that used HRT longer than 5 years. In the elderly cohort, there were 0.7 additional cases of breast cancer for every 1000 person-years of HRT use. The population-based risk of breast cancer attributable to HRT use in the elderly cohort was 3.1%.

## 4. Discussion

Compared with the older generation of women, younger women in Taiwan have a higher fat intake, increased obesity, earlier menarche, delayed age of childbirth, increased nulliparity, and less breast feeding, which causes the birth-cohort difference in the risk of breast cancer [16]. Although the above risk factors were not present in the claim data, the data was adjusted for the summated effect of these factors. The lack of individual demographic risk factors in the claim data is a major limitation of this study. Nevertheless, the implementation of NHI has reduced the socioeconomic barrier to HRT use, and women with a higher pre-existing risk of breast cancer were less prone to receive HRT in clinical practice [21]. Furthermore, HRT users in our data did not show pre-existing higher breast cancer HR *versus* non-users in the initial three years of follow-up (Table 2).

From a literature review, past HRT users had a lower risk of breast cancer than did current users because the risk dissipates after the discontinuation of HRT use [3,6,20]. This explains why a recent systemic review in Korea showed no significant effect of HRT history on the risk of breast cancer [8], where HRT history represented only past, not current, use. In our study, HRT users were censored after the discontinuation of HRT to avoid neutralization of the results from past users.

Compatible with the results of the Women’s Health Initiatives (WHI) study [1,2], our study found that long-term use of estrogen plus progestin increased the risk of breast cancer in women 45–64 years of age, but estrogen alone did not. Women on estrogen alone in our population had mostly received hysterectomy and oophorectomy, which may reduce intrinsic estrogen exposure. Progestin may play a major role in inducing breast cancer when combined with estrogen [22,23,24]. The role of progestin in the pathogenesis of breast cancer deserves further investigation [25].

The use of HRT in Taiwan has declined since 2002 following the publication of the WHI results [26,27]; hence, the impact of HRT on the population-based risk of breast cancer is decreasing. However, breast cancer incidence is still increasing [17]. Other environmental endocrine disruptors, such as plasticizers, deserve further surveillance and investigations.

## 5. Conclusions

More than four years of current HRT use can increase the risk of breast cancer in Asian women, independently of the birth-cohort difference. A longer duration of current HRT use is associated with a higher risk of breast cancer. Estrogen combined with progestin significantly increases the risk of breast cancer in middle-aged women, but not the use of estrogen alone.

## Figures and Tables

**Table 1:** The breast cancer incidence of current hormone replacement therapy (HRT) users and non-users from 1997 to 2004.

	Person	Observed Person-Years	Breast Cancer Incident Cases	Breast Cancer Incident Rate (Cases Per 100,000 Person-Years)	HR ^a^ (95% CI)
Women aged 45–64 years in 1997					
Non-HRT users	12,466	96,246	147	153	1
Current HRT users	3397	11,371	34	299	1.95 (1.32–2.87)
Estrogen only	873	3195	7	219	1.37 (0.64–2.95)
Estrogen plus progestin	2524	8176	27	330	2.17 (1.41–3.32)
Sequential estrogen plus progestin	829	2631	10	380	2.40 (1.25–4.61)
Continuous estrogen plus progestin	1695	5545	17	307	1.98 (1.18–3.32)
Women aged ≥65 years in 1997					
Non-HRT users	6516	44,385	34	77	1
Current HRT users	550	1412	2	142	1.85 (0.43–7.90)

^a^: Hazard ratio by Cox proportional hazards model; CI: confident interval. The HRT regimen included all estrogen and progestin types in oral or topical forms.

**Table 2:** Breast cancer hazard ratio of HRT users *versus* non-users by Cox proportional hazards model, adjusted for birth cohort.

Follow-Up Time * (Years)	HR of Breast Cancer	95% CI	*p* Value
1	0.91	0.63–1.30	0.593
2	1.10	0.76–1.58	0.617
3	1.28	0.89–1.84	0.186
4	1.48	1.03–2.13	0.035
5	1.65	1.14–2.39	0.007
6	1.83	1.26–2.65	0.001
7	1.92	1.32–2.79	0.001
8	1.95	1.34–2.84	<0.001

*: The follow-up time for HRT users was the duration of HRT use because they were censored after discontinuation of HRT.
